# Immunohistochemical Profiling of SSTR2 and HIF-2α with the Tumor Microenvironment in Pheochromocytoma and Paraganglioma

**DOI:** 10.3390/cancers16122191

**Published:** 2024-06-11

**Authors:** Masaki Uchihara, Akiyo Tanabe, Yuki Kojima, Tatsunori Shimoi, Akiko Miyagi Maeshima, Kotaro Umamoto, Akihiko Shimomura, Chikako Shimizu, Yuto Yamazaki, Eijiro Nakamura, Yoshiyuki Matsui, Nobuyuki Takemura, Hideyo Miyazaki, Kazuki Sudo, Kan Yonemori, Hiroshi Kajio

**Affiliations:** 1 Department of Diabetes, Endocrinology and Metabolism, National Center for Global Health and Medicine, Toyama 1-21-1, Shinjuku-ku City, Tokyo 162-8655, Japan; muchihara@hosp.ncgm.go.jp (M.U.);; 2 Course of Advanced and Specialized Medicine, Juntendo University Graduate School of Medicine, 3-1-3 Hongoh, Bunkyo-ku, Tokyo 113-0033, Japancshimizu@hosp.ncgm.go.jp (C.S.); 3 Department of General Internal Medicine, Oncological Endocrinology, National Cancer Center Hospital, Tsukiji 5-1-1, Chuo-ku, Tokyo 104-0045, Japan; 4 Department of Medical Oncology, National Cancer Center Hospital, Tsukiji 5-1-1, Chuo-ku, Tokyo 104-0045, Japantshimoi@ncc.go.jp (T.S.);; 5 Department of Diagnostic Pathology, National Cancer Center Hospital, Tsukiji 5-1-1, Chuo-ku, Tokyo 104-0045, Japan; 6 Department of Breast and Medical Oncology, National Center for Global Health and Medicine, Toyama 1-21-1, Shinjuku-ku City, Tokyo 162-8655, Japan; 7 Department of Pathology, Tohoku University Graduate School of Medicine, Seiryo-machi 2-1, Aoba-ku, Sendai, Miyagi 980-8575, Japan; 8 Department of Urology, National Cancer Center Hospital, Tsukiji 5-1-1, Chuo-ku, Tokyo 104-0045, Japan; 9 Hepato-Biliary-Pancreatic Surgery Division, Department of Surgery, National Center for Global Health and Medicine, Toyama 1-21-1, Shinjuku-ku City, Tokyo 162-8655, Japan; 10 Department of Urology, National Center for Global Health and Medicine, Toyama 1-21-1, Shinjuku-ku City, Tokyo 162-8655, Japan

**Keywords:** pheochromocytoma, paraganglioma, immunotherapy, somatostatin receptor, tumor microenvironment, HIF2α

## Abstract

**Simple Summary:**

Metastatic pheochromocytomas and paragangliomas (PPGLs) are rare endocrine malignancies with limited effective treatment options. The association between the tumor microenvironment with somatostatin receptor 2 (SSTR2) and hypoxia-induced factor-2α (HIF-2α) in PPGLs, critical for optimizing combination therapeutic strategies with immunotherapy, remains largely unexplored. We found associations between tumor-associated macrophages and SSTR2A expression, and between PD-L1 and HIF-2α expression. Our data suggests the potential of combination therapy with immunotherapy and peptide receptor radionuclide therapy or HIF-2α inhibitors as a treatment option in selected PPGL populations.

**Abstract:**

Metastatic pheochromocytomas and paragangliomas (PPGLs) are rare endocrine malignancies with limited effective treatment options. The association between the tumor microenvironment (TME) with somatostatin receptor 2 (SSTR2) and hypoxia-induced factor-2α (HIF-2α) in PPGLs, critical for optimizing combination therapeutic strategies with immunotherapy, remains largely unexplored. To evaluate the association of SSTR2 and HIF-2α immunoreactivity with the TME in patients with PPGLs, we analyzed the expression of SSTR2A, HIF-2α, and TME components, including tumor-infiltrating lymphocytes (CD4 and CD8), tumor-associated macrophages (CD68 and CD163), and PD-L1, using immunohistochemistry in patients with PPGLs. The primary outcome was to determine the association of the immune profiles with SSTR2A and HIF-2α expression. Among 45 patients with PPGLs, SSTR2A and HIF2α were positively expressed in 21 (46.7%) and 14 (31.1%) patients, respectively. The median PD-L1 immunohistochemical score (IHS) was 2.0 (interquartile range: 0–30.0). Positive correlations were observed between CD4, CD8, CD68, and CD163 levels. A negative correlation was found between the CD163/CD68 ratio (an indicator of M2 polarization) and SSTR2A expression (r = −0.385, *p* = 0.006). HIF-2α expression showed a positive correlation with PD-L1 IHS (r = 0.348, *p* = 0.013). The co-expression of PD-L1 (HIS > 10) and HIF-2α was found in seven patients (15.6%). No associations were observed between SDHB staining results and the CD163/CD68 ratio, PD-L1, or SSTR2A expression. Our data suggest the potential of combination therapy with immunotherapy and peptide receptor radionuclide therapy or HIF-2α inhibitors as a treatment option in selected PPGL populations.

## 1. Introduction

Pheochromocytomas and paragangliomas (PPGLs) are rare neuroendocrine tumors with up to 20% of cases being metastatic (mPPGLs) [1]. Most current treatments for mPPGLs have limited efficacy, or they become resistant over time, emphasizing the critical need to develop new therapies [2]. Immunotherapy, a breakthrough in oncology, has been shown to be effective in some cases of mPPGLs; however, the response rate remains limited [3]. Therefore, one strategy to improve immunotherapy is combining drugs with different action mechanisms and target resistance [4,5].

The analysis of the tumor microenvironment (TME), like programmed death ligand 1 (PD-L1) and tumor-associated macrophages (TAMs), offers insights into resistance to immunotherapy [6]. Macrophages are highly plastic and heterogeneous, polarizing in response to cytokine interactions [7]. While proinflammatory M1 macrophages play a role in anti-tumor activities, anti-inflammatory M2 macrophages promote tumor progression and immune evasion, reducing sensitivity to immunotherapy [8,9,10]. The prevalence of M2-polarized TAMs in mPPGLs [11,12,13] underscores the potential of targeting M2 polarization in macrophage-specific therapies, which involve repolarizing M2 macrophages to M1 or depleting them [14]. Immunohistochemistry (IHC) evaluation of the CD163/CD68 ratio allows the assessment of M2 polarization as an effective and cost-efficient method.

Radiation and radionuclide therapy have emerged as promising strategies to enhance immune checkpoint inhibitor (ICI) efficacy by modulating the TME. These alterations encompass changes in the expression of cell-surface antigens [15] and impact macrophage polarization [16]. Some PPGLs have exhibited the abscopal effect of external radiation, as initially reported by our institute [17], suggesting that radiotherapy could activate an anti-tumor immune response [18]. The potential of combining ICIs with peptide receptor radionuclide therapy (PRRT) targeting somatostatin receptor 2 (SSTR2) has been demonstrated in basic research in animal models of gastrointestinal neuroendocrine neoplasms (GEP-NENs) [4] and clinical cases of Merkel cell carcinoma and pituitary carcinoma [19,20]. As SSTR2 is relatively highly expressed in PPGLs [21,22,23], PRRT is currently being evaluated in clinical trials [2]. However, to our knowledge, the interaction between SSTR2 and the PPGLs tumor microenvironment for potential combined ICIs and PRRT therapy remains largely unexplored. Our study aims to fill this gap, suggesting new directions for therapeutic research in PPGLs. 

Hypoxia-related pathways also promote immune tolerance in the immune cell components [24]. HIF-2α expression in renal cell carcinoma (RCC) correlates with increased PD-L1 expression in tumors, indirectly suppressing T-cell function [25]; hence, HIF-2α inhibitors are being investigated in combination with ICIs in RCC [26]. The stabilization of HIF-2α in PPGLs is gaining attention as a promising therapeutic target [27]. Clinical trials of Belzutifan (MK-6482), a selective HIF-2α inhibitor approved for von Hippel–Lindau (VHL) disease-associated tumors (RCC, pancreatic neuroendocrine tumor (NET), and central nervous system hemangioblastoma) [28], have expanded to PPGLs (NCT04924075). Although some PPGLs have pathogenic mutations in genes associated with hypoxia signaling, there are limited reports on the association between the immune cell components and HIF expression [29,30,31].

As the development of combined immunotherapies progresses in mPPGLs, the heterogeneity of the TME necessitates the assessment of the immune profiles of each tumor [11,13,32]. Therefore, our study aimed to investigate the immunoreactivity and interplay of SSTR2, HIF-2α, and TME in PPGLs.

## 2. Material and Methods

### 2.1. Patients and Samples

We analyzed 45 patients diagnosed with PPGLs and preserved FFPE (formalin-fixed, paraffin-embedded) tissue blocks at the National Center for Global Health and Medicine or the National Cancer Center Hospital in Japan. Inclusion criteria were diagnosis between 1988 and 2020, and a pathological diagnosis of PPGL. Exclusion criteria included the absence of a PPGL diagnosis, lack of preserved FFPE specimens, or refusal of consent. The clinicopathological and gene mutation data for each patient were gathered from medical records. Among six patients who underwent genetic testing, three had known germline mutations of PPGL susceptibility genes (1 SDHB, 1 SDHD, 1 VHL). The remaining patients had not undergone genetic testing at the time of data collection; therefore, no genetic data are available for their tumors. Metastatic events were defined as distant metastases in non-chromaffin tissues. The tumor samples consisted of 43 primary tumors and 10 metastases. In cases with available paired primary-metastasis samples, only one tumor per patient was used for analysis (primary tumor for baseline and predictive analysis, metastases for correlation analysis with other biomarkers).

The study protocol conformed to the ethical guidelines of the 1975 Declaration of Helsinki and was approved by the Institutional Review Board of the National Center for Global Health and Medicine, Tokyo, Japan (approval number: NCGM-S-004328-00). Due to the retrospective, non-interventional nature of the study, an opt-out model was employed for patient consent.

### 2.2. IHC and Its Evaluation

Hematoxylin and eosin slides were reviewed to identify pathological features. The slides were obtained by cutting the archived FFPE tissue blocks into serial 5-µm sections. Antibodies targeting SSTR2A (AB_2737601, Abcam, Cambridge, UK, 1:2000), SDHB (AB_301432, Abcam, Cambridge, UK, 1:1000), HIF-2α (AB_10002593, Novus Biologicals, Centennial, CO, USA, 1:100) were used for immunostaining. To evaluate immune cell components, antibodies targeting tumor-infiltrating lymphocytes (CD4 [AB_876941, Leica, Wetzlar, Germany, 1:50] and CD8 [AB_10555292, Leica, Wetzlar, Germany, 1:50]), tumor-associated macrophages (CD68 [AB_2074844, Dako, Glostrup, Denmark, 1:500] and CD163 [AB_2756375, Leica, Wetzlar, Germany, 1:200]), PD-L1 (AB_2833074, Dako, Glostrup, Denmark, Ready for use), and Ki-67 (AB_2631211, Dako, Glostrup, Denmark, 1:100) were used. The specific protocols for each marker are detailed in Table S1 Supplementary Materials.

The immunoreactivity of SSTR2A was evaluated based on Volante scores [21,33], considering the subcellular localization and extent of the staining in four levels: 0 (no staining), 1 (pure cytoplasmic immunoreactivity, either focal or diffuse), 2 (membranous reactivity in less than 50% of tumor cells, irrespective of the presence of cytoplasmic staining), 3 (circumferential membranous reactivity in more than 50% of tumor cells, irrespective of the presence of cytoplasmic staining). HIF-2α was evaluated in four levels of staining: 0 (no staining), 1 (weak), 2 (medium), and 3 (strong) [34], each for the tumor cell nuclear (HIF-2α^NUC^) and cytoplasm (HIF-2α^CYT^) based on previous reports in PPGLs [31,35]. For statistical analysis, SSTR2A and HIF-2α scores of 0 and 1 were considered negative, while scores of 2 and 3 were considered positive. Positive SDHB staining was determined by granular solid staining in the cytoplasm, while negative staining was indicated by the absence of such staining despite positive staining in internal control sustentacular cells. A distinct subgroup was identified for tumors displaying a cytoplasmic blush without clear granularity, categorized as a weak-diffuse pattern [36]. Based on the genetic finding of succinate dehydrogenase gene mutations in two of the three cases with weak-diffuse patterns (one case untested), these populations were included in the negative SDHB staining group for statistical purposes. The Ki-67 labeling index (Ki-67 LI) was evaluated after identifying the hot spot of the whole tumor. PD-L1 expression was assessed by the semi-quantitative immunohistochemical score (IHS). Positive PD-L1 results were defined by positive tumor cell membrane or cytoplasm staining. The integrating staining intensity was graded on a scale from 0 to 3: 0 (no staining), 1 (weak), 2 (medium), and 3 (strong). The proportion of positive tumor cells was evaluated, scoring from 0% to 100%. The final IHS was calculated by multiplying the staining intensity and the proportion of positive tumor cells, yielding a range from 0 to 300. An IHS of 10 or more was classified as positive [37]. CD68 and CD163 were used as markers for total infiltrating macrophages and M2-polarized macrophages, respectively [38]. The CD163/CD68 ratio was calculated to assess M2-polarization. Nuclear immunoreactivity of CD4, CD8, CD68, and CD163 was assessed by the proportion of positive cells in the tumor parenchyma (38). We used normal adrenal medullary tissue obtained from non-PPGL cases as controls in the pathological assessments. The evaluation was performed by two pathologists blinded to the clinical data.

### 2.3. Statistical Analysis

The Kruskal–Wallis, chi-square, and Mann–Whitney *U* tests, where appropriate, were used to compare clinicopathological parameters between the groups. Spearman’s rank test was performed for correlation analysis. Disease progression was defined as an increase of more than 20% in the size of the primary or metastatic lesions or the appearance of new tumors [39]. Time to progression (TTP) was measured from confirmed PPGL diagnosis to first disease progression. Metastasis-free survival (MFS) was defined as the duration between the surgery date and the detection of metastasis. The Kaplan–Meier method was used to calculate the median TTP and MFS. A *p*-value of less than 0.05 was considered statistically significant. Statistical analyses were performed using GraphPad Prism version 9.5 (GraphPad Software Inc., San Diego, CA, USA) and EZR version 1.67 (Saitama Medical Center, Jichi Medical University, Saitama, Japan) [40], which is a graphical user interface for R (version 4.2, The R Foundation for Statistical Computing, Vienna, Austria).

## 3. Results

### 3.1. Patient Characteristics

The clinicopathologic features of the patient cohort are shown in Table 1. The median age at diagnosis was 46 years (range: 17–80 years). Of the 45 patients, 28 were female (62.2%). The primary tumor was located in the adrenal gland in 18 cases and in the extra-adrenal gland in 27 cases. The median follow-up duration was 50 months (range: 2–407 months). Among the 18 patients with mPPGLs, 10 had distant metastases at the time of primary tumor diagnosis, and eight developed systemic recurrence during follow-up. Factors associated with metastatic behavior included younger age, extra-adrenal primary tumor location, negative 123I-MIBG uptake, negative SDHB staining, high Ki-67 LI (≥3%), and larger tumor size (≥50 mm) (*p* < 0.05). All patients did not undergo embolization prior to surgery or biopsy.

### 3.2. SSTR2A and HIF-2α Expression with Clinicopathological Profile 

**Figure 1:**
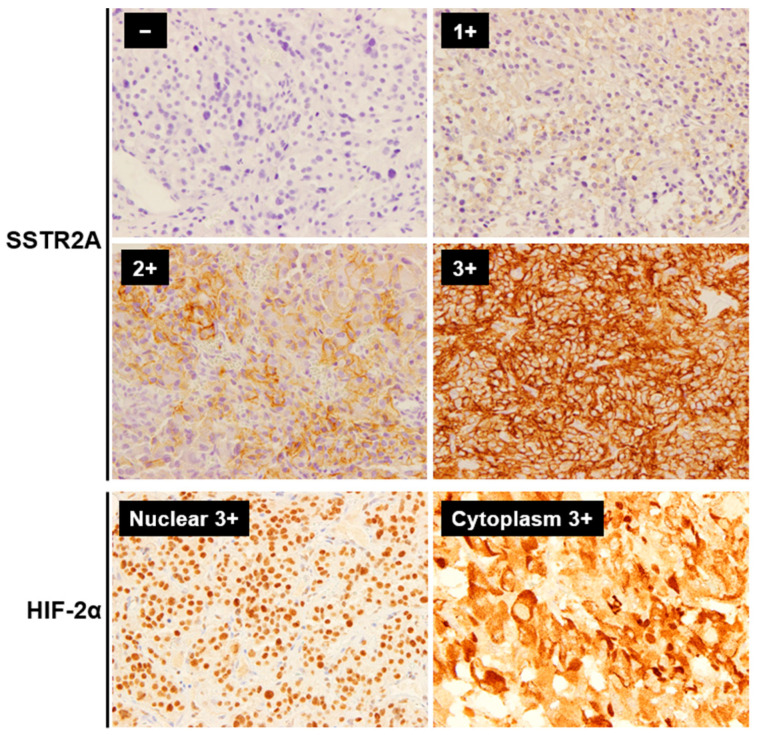
Representative immunohistochemistry staining images of SSTR2A and HIF-2α in PPGLs (Magnification: ×400). The immunoreactivity of SSTR2A was evaluated based on Volante scores. For statistical analysis, SSTR2A scores of 0 and 1 were considered negative, while 2 and 3 were considered positive. HIF-2α was evaluated for both the tumor cell nucleus and cytoplasm. Abbreviations: SSTR2A, somatostatin receptor 2A; HIF, hypoxia-induced factor.

SSTR2A and HIF-2α expressions were evaluated in 50 tumor samples from 45 patients (Figure 1 and Figure S1). The positive expression of SSTR2A (SSTR2A^+^) was observed in 21 patients (46.7%). Among five patients who underwent 111In-Pentetreotide scintigraphy, two with uptake were in the SSTR2A^+^ group, and three without uptake were in the SSTR2A^−^ group. SSTR2A positivity was associated with younger ages, metastatic tumors (12/21 [57.1%] vs. 6/24 [25.0%]), and higher Ki-67 LI (Table 2), while the length of the TTP was not significantly different based on SSTR2A status. No correlation was observed between SSTR2A positivity and SDHB staining results. The positive staining of HIF-2α was exclusively localized in the nucleus and cytoplasm (Table S2), and 14 patients (31.1%) were HIF-2α^NUC^ positive. Metastatic tumors, high Ki-67 LI, negative 123I-MIBG uptake, and negative SDHB staining were associated with HIF-2α^NUC^-positivity (*p* < 0.05). All primary tumors located in the adrenal gland tested negative for HIF-2α^NUC^. In the analyses for the 27 patients with primary extra-adrenal tumors, high Ki-67 LI and negative SDHB staining were associated with HIF-2α^NUC^-positivity, whereas metastatic behavior and 123I-MIBG uptake were not. In the five patients with available pairs of primary and metastatic sites, the positivity of SSTR2A and HIF2α^NUC^ were all consistent between primary and metastatic tumor samples. The results of the comparative analysis of SSTR2A and HIF-2α expression across four groups are detailed in Table S3. The group with both negative SSTR2A and HIF-2α^NUC^ expression showed fewer examples of metastatic behavior, lower Ki-67 LI, predominantly positive MIBG uptake, and mostly positive SDHB staining compared to the other groups.

### 3.3. TME with SSTR2A and HIF-2α Expression

**Figure 2:**
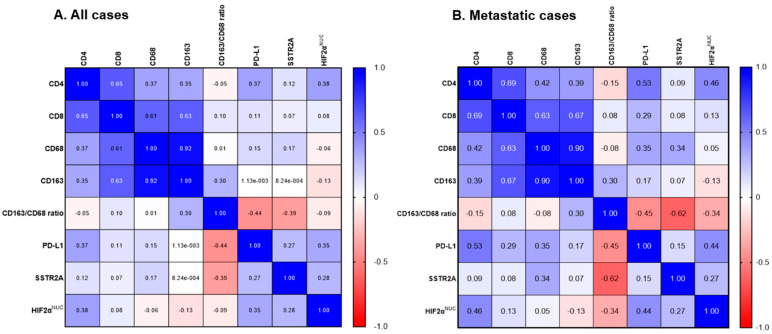
Correlation matrix of SSTR2A and HIF-2α with tumor microenvironment in PPGLs. (**A**) All patients (*n* = 45). (**B**) Metastatic cases (*n* = 18). Abbreviations: SSTR2A, somatostatin receptor 2A; HIF, hypoxia-induced factor; NUC, nuclear.

**Figure 3:**
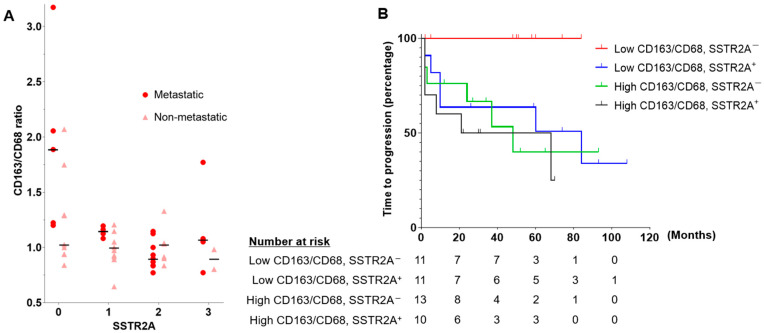
(**A**) Scatterplot showing a positive correlation between SSTR2A expression and CD163/CD68 ratio. The horizontal bars indicate the median CD163/CD68 ratio for each SSTR2A score in metastatic and non-metastatic PPGLs. (**B**) The time to progression analyses according to CD163/CD68 ratio and SSTR2A expression in patients with PPGLs. Abbreviations: SSTR2A, somatostatin receptor 2A.

The median levels (interquartile range) of PD-L1, CD4, CD8, CD68, and CD163 were 2.0 (0–30.0), 0.6 (0.1–1.2), 1.2 (0.8–2.1), 9.8 (5.2–12.8), and 8.8 (6.2–14.4), respectively. Representative IHC images are shown in Figure S2. PD-L1 positivity (IHS > 10) was noted in 17 tumors (37.8%). The median CD163/CD68 ratio was 1.03 (range: 0.65–3.18), used as a cut-off value to classify patients into high and low groups. There was no significant association between PD-L1 positivity, high CD163/CD68 ratio, and clinicopathological characteristics, including age, sex, primary tumor location, metastatic behavior, and SDHB staining (Table S4). Positive correlations were noted among CD4, CD8, CD68, and CD163 levels (Figure 2A). There was a negative correlation between CD163/CD68 ratio and SSTR2A expression (r = −0.385, *p* = 0.006), with a stronger correlation in metastatic cases (r = −0.535, *p* = 0.009) (Figure 2 and Figure 3A). No correlation was found between SSTR2A expression and other immune cell components.

In light of the negative correlation observed between SSTR2A and the CD163/CD68 ratio, our analysis assessed the TTP and MFS across the four groups based on these parameters (Figure 3B and Figure S3, Table S5). Although no significant differences were observed overall, all 11 patients in the low CD163/CD68 ratio and SSTR2A^−^ group showed no progression or detection of metastasis.

HIF-2α^NUC^ expression was positively correlated with PD-L1 IHS (r = 0.348, *p* = 0.013), particularly in metastatic cases (r = 0.441, *p* = 0.035) (Figure 2A,B). The co-expression of PD-L1 (IHS > 10) and HIF-2α^NUC^ was observed in 15.6% (7/45) of the entire cohort and 25.9% (7/27) of patients with primary extra-adrenal tumors. Among those with positive PD-L1 expression, 41.1% (7/17) also showed positive HIF-2α^NUC^ expression.

## 4. Discussion

In this study, we found a negative correlation between M2 macrophage polarization (indicated by the CD163/CD68 ratio) and SSTR2A expression in PPGLs, especially in metastatic cases. Additionally, we noted that high PD-L1 expression was linked with increased HIF-2α^NUC^ expression. Hence, our data indicates the potential for combination immunotherapy strategies.

We found an inverse relationship between M2 macrophage polarization and SSTR2 expression in PPGLs. This research represents an initial understanding of the relationship between SSTR2 expression and immune cell infiltration in PPGLs. Our findings suggest a foundation upon which further studies might build to explore innovative treatments. Considering the tendency of M2-polarized tumors to exhibit resistance to immunotherapy, alongside the effectiveness of PRRT in tumors with high SSTR2 expression, our findings indicate that a large group of patients could be potential candidates for combined therapy of PRRT with ICIs, including pembrolizumab, nivolumab, and ipilimumab, similar to other NETs currently under study [4,19,20]. Given the overexpression of SSTR2 in B cells, PRRT causes a temporary selective reduction in B cells without significantly affecting T cells, which is unlikely to hinder the effectiveness of ICIs [41]. Temozolomide, sunitinib, and PARP inhibitors, which are therapeutic or candidate drugs for PPGLs, have been reported to upregulate SSTR in other cancer types [42]. In addition, some reports showed that SSTR is also present in macrophages; furthermore, the somatostatin analog could alter macrophage functions [43,44]. Based on our results, future research should focus on how changes in macrophage polarity and other TME components occur in PPGLs treated with therapies that upregulate SSTR2A or radionuclide therapy. Notably, not all patients in the group with low CD163/CD68 ratios and negative SSTR2A showed disease progression. Although a multivariate analysis was not feasible due to the limited sample size, these markers could be worth exploring as a potential prognostic factor in future analysis.

We observed that high PD-L1 expression was associated with high HIF-2α^NUC^ expression in PPGLs. Hypoxia-related pathways, which enhance PD-L1 expression in cancer cells, contribute to immune tolerance in the TME [24,45]. Previous studies on the relationship between PD-L1 and HIF-α expression in PPGLs were insufficient as only HIF-1α was assessed [29], not distinguishing between nuclear and cytoplasmic HIF-2α staining [30] or only in head and neck paragangliomas with prior embolization [31]. Although further research, particularly transcriptome analysis, is necessary to validate the IHC findings of HIF-2α, the elevated levels of both PD-L1 and HIF-2α observed in our diverse cohorts indicate a potential approach for combination therapy using HIF-2α inhibitors and ICIs.

Approximately 30–35% of PPGLs have germline mutations [46], with SDHB mutation being common and associated with metastasis [47,48]. Immunostaining for lack of SDHB expression is a clinically accessible and cost-effective method for identifying patients likely to carry SDHx mutations [49,50]. Our study did not show an association between SDHB staining results and M2 macrophage polarization or SSTR2A expression. Previous studies have reported the association of M2 polarization with SDHB mutation [3,4]; however, other studies have shown the opposite association with the kinase signaling subtype [11], highlighting the contradictory nature of the current literature. Studies on the expression of SSTR2A and the results of SDHB staining have been mixed, with some showing high SSTR2A expression in cases of negative SDHB staining [22], while others report low SSTR2A expression [51]; recent research indicates no association between SDHB staining and SSTR2A expression [21]. Our data also show no correlation, suggesting that further investigations, such as immunohistochemistry, functional imaging, and genetic testing, are needed to clarify their relationship [52,53].

This study had some limitations. First, our study evaluated a limited number of tumor samples, a challenge often faced in studying rare diseases. This limitation may restrict the extrapolation of our findings to the broader PPGL population, necessitating that our results be viewed as hypothesis-generating rather than conclusive. The limited observation period also restricted our ability to assess long-term overall survival. Future studies with larger cohorts and extended follow-up periods are necessary to validate these findings. However, given the scarce data in this field, sharing our results could still aid ongoing research efforts for therapeutic developments. Second, genetic testing is not widely performed in Japan due to its high cost and lack of insurance coverage. This study could not analyze FFPE specimens due to the difficulty of maintaining the condition of old FFPE specimens for genetic analysis. Consequently, the number of patients who had undergone germline genetic testing was limited. Finally, the specificity of CD163 in evaluating M2 macrophages remains a challenge, necessitating cautious interpretation of its expression. Since many markers could evaluate M2 macrophages, CD163 cannot cover all M2 macrophage subtypes. To further improve specificity and enhance our findings’ reliability, we plan to incorporate additional markers such as CD206 and CD204 and utilize multiplex immunofluorescence methods in future studies.

## 5. Conclusions

In conclusion, we found a negative association between M2 macrophage polarization and SSTR2A expression in PPGLs. We also observed a relationship between high PD-L1 expression and increased HIF-2α^NUC^ expression. Our data indicate the potential of using a combination of immunotherapy with PRRT or HIF-2α inhibitors as a therapeutic avenue in selected PPGL populations; however, due to the small sample size and limited genetic background data, these results should be considered hypothesis-generating. Future large-scale studies where all patients undergo germline testing are required to validate our findings further.

## Figures and Tables

**Table 1:** Patient Characteristics.

	Metastatic PPGL	Non-Metastatic PPGL	*p*
	(*n* = 18)	(*n* = 27)	
**Age at Initial Diagnosis** **(median, range)**	39 (18–65)	58 (17–80)	0.01
**Sex**			
Female	10	18	0.537
Male	8	9	
**Primary Tumor Location**			
Adrenal	3	15	0.012
Extra-adrenal	15	12	
Abdominal	10	10	
Head and neck	2	0	
Bladder	3	2	
**Functional Status**			
Adrenergic	1	12	0.012
Noradrenergic	8	4	
Silent or Dopaminergic	7	9	
Not available	2	2	
**Diabetes Mellitus**	7	5	0.175
**Hypertension**	10	15	1
**Ki-67 LI**			
≥3%	14	9	0.006
<3%	4	18	
**Tumor Size**			
≥50 mm	14	13	0.065
<50 mm	4	14	
**123I-MIBG Uptake**			
Positive	9	24	0.007
Negative	6	1	
Not available	3	2	
**SDHB Staining**			
Positive	6	26	<0.001
Negative	12	1	

Abbreviations: PPGL, pheochromocytoma and paraganglioma; Ki-67 LI, Ki-67 labeling index; MIBG, Metaiodobenzylguanidine; SDHB, succinate dehydrogenase subunit B.

**Table 2:** Characteristics of SSTR2A and HIF-2α expression in PPGLs.

	SSTR2A		*p*	HIF-2α^NUC^		*p*
	Positive	Negative		Positive	Negative	
	(*n* = 21)	(*n* = 24)		(*n* = 14)	(*n* = 31)	
**Age at Initial Diagnosis** **(median, range)**	40 (17–70)	59 (18–80)	0.005	41 (17–70)	50 (18–80)	0.333
**Sex**						
Female	15	13	0.356	7	21	0.326
Male	6	11		7	10	
**Primary Tumor Location**						
Adrenal	7	11	0.599	0	18	<0.001
Extra-adrenal	14	13		14	13	
Abdominal	10	10		11	9	
Head and neck	2	0		1	1	
Bladder	2	3		2	3	
**Tumor Site**						
Adrenal	7	10	0.704	0	17	0.001
Abdominal	10	9		10	9	
Liver	0	1		1	0	
Head and neck	2	1		1	2	
Bladder	2	3		2	3	
**Functional Status**						
Adrenergic	6	7	0.743	1	12	0.060
Noradrenergic	7	5		3	9	
Silent or Dopaminergic	7	9		8	8	
Not available	1	3		2	2	
**Metastatic PPGL**						
Yes	12	6	0.037	9	9	0.047
No	9	18		5	22	
**Ki-67 LI**						
≥3%	15	8	0.017	13	10	<0.001
<3%	6	16		1	21	
**GAPP Score**						
≥7	7	5	0.274	5	7	0.658
3–7	13	14		7	20	
<3	1	5		2	4	
**123I-MIBG Uptake**						
Positive	14	19	0.226	7	26	0.003
Negative	5	2		6	1	
Not available	2	3		1	4	
**SDHB Staining**						
Positive	12	20	0.098	4	28	<0.001
Negative	9	4		10	3	

Abbreviations: SSTR2A, somatostatin receptor 2A; HIF, hypoxia-induced factor; NUC, nuclear; PPGL, pheochromocytoma and paraganglioma; Ki-67 LI, Ki-67 labeling index; MIBG, Metaiodobenzylguanidine; SDHB, succinate dehydrogenase subunit B; GAPP, Grading of Adrenal Pheochromocytoma and Paraganglioma.

## Data Availability

The datasets are not publicly available but are available from the corresponding author upon reasonable request.

## Supplementary Materials

The following supporting information can be downloaded at: https://www.mdpi.com/article/10.3390/cancers16122191/s1, Table S1: Antibodies used for immunohistochemistry; Table S2: The characteristics of HIF-2α expression in PPGLs; Table S3: Characteristics and group comparisons of SSTR2A and HIF-2α expression in PPGLs; Table S4: Characteristics of PD-L1 expression and CD163/CD68 ratio in PPGLs; Table S5: Characteristics and group comparisons of CD163/CD68 ratio and SSTR2A expression in PPGLs; Figure S1: Representative immunohistochemistry staining images of SSTR2A in PPGLs; Figure S2: Representative immunohistochemistry staining images of SDHB, CD8, CD68, CD163, and PD-L1 in PPGLs (Magnification: ×400, except for CD8 at ×200); Figure S3: The metastatic free survival analyses according to CD163/CD68 ratio and SSTR2A expression in patients with PPGLs.
