# Supplementary material for: Immunohistochemical Profiling of SSTR2 and HIF-2α with the Tumor Microenvironment in Pheochromocytoma and Paraganglioma
Source: Cancers (Basel). 2024 Jun 11;16(12):2191. doi: 10.3390/cancers16122191 (PMC11201597; doi:10.3390/cancers16122191)
Supplement: Supplementary file 1 [file cancers-16-02191-s001.zip › cancers-3016936-supplementary.pdf]

Table S1. Antibodies used for immunohistochemistry.

Antibody	Clone	Identifiers (RRID)	Type/Host	Company	Dilution
SSTR2A	UMB-1	AB_2737601	Rabbit monoclonal	Abcam	1:2000
SDHB	21A11	AB_301432	Mouse monoclonal	Abcam	1:1000
HIF-2α	NB100-122	AB_10002593	Rabbit polyclonal	Novus Biologicals	1:100
CD4	1F6	AB_876941	Mouse monoclonal	Leica	1:50
CD8	4B11	AB_10555292	Mouse monoclonal	Leica	1:50
CD68	PG-M1	AB_2074844	Mouse monoclonal	Dako	1:500
CD163	10D6	AB_2756375	Mouse monoclonal	Leica	1:200
PD-L1	22C3	AB_2833074	Mouse monoclonal	Dako	Ready for use
Ki-67	MIB-1	AB_2631211	Mouse monoclonal	Dako	1:100

Abbreviations: RRID, Research Resource Identifier; SSTR, somatostatin receptor; SDH, succinate dehydrogenase; HIF, hypoxia-induced factor; PD-L1, programmed cell death ligand 1.

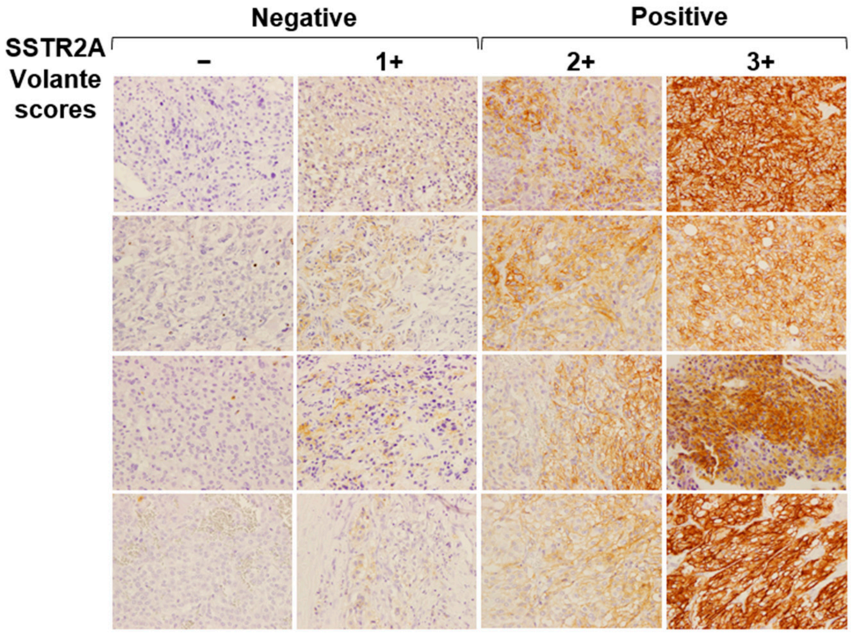

Figure S1. Representative immunohistochemistry staining images of SSTR2A in PPGLs (Magnification: ×400). The immunoreactivity of SSTR2A was evaluated based on Volante scores. For statistical analysis, SSTR2A scores of 0 and 1 were considered negative, while 2 and 3 were considered positive. Abbreviations: SSTR2A, somatostatin receptor 2A.

Table S2. Characteristics of HIF-2α expression in PPGLs.

		HIF-2α^{NUC}	
		Positive	Negative
HIF-2α^{CYT}	Positive	3	30
	Negative	11	6

Abbreviations: PPGL, pheochromocytoma and paraganglioma; HIF, hypoxia-induced factor; NUC, nuclear; CYT, cytoplasm.

Table S3. Characteristics and group comparisons of SSTR2A and HIF-2α expression in PPGLs.

	SSTR2A Negative, HIF2αNUC Negative (n = 19)	SSTR2A Positive, HIF2αNUC Negative (n = 12)	SSTR2A Negative, HIF2αNUC Positive (n=5)	SSTR2A Positive, HIF2αNUC Positive (n=9)	*P*
**Age at Initial Diagnosis (median, range)**	58 (18-80)	42.5 (27-59)	62 (35-65)	36 (17-70)	0.043
**Sex**					
Female	11	10	2	5	0.302
Male	8	2	3	4	
**Primary Tumor Location**					
Adrenal	11	7	0	0	0.072
Extra-adrenal	8	5	5	9	
Abdominal	6	3	4	7	
Head and neck	0	1	0	1	
Bladder	2	1	1	1	
**Tumor Site**					
Adrenal	10	7	0	0	0.029
Abdominal	6	3	3	7	
**Liver**	0	0	1	0	
Head and neck	1	1	0	1	
Bladder	2	1	1	1	
**Functional Status**					
Adrenergic	7	5	0	1	0.357
Noradrenergic	4	5	1	2	
Silent or Dopaminergic	6	2	3	5	
Not available	2	0	1	1	
**Metastatic PPGL**					
Yes	3	6	3	6	0.034
No	16	6	2	3	
**Ki-67 LI**					
≥ 3%	3	7	5	8	<0.001
< 3%	16	5	0	1	
**GAPP Score**					
≥ 7	3	4	2	3	0.558
3-7	12	8	2	5	
< 3	4	0	1	1	
**123I-MIBG Uptake**					
Positive	16	10	3	4	0.005

Negative	1	0	1	5	
Not available	2	2	1	0	
**SDHB Staining**					
Positive	18	10	2	2	<0.001
Negative	1	2	3	7	

Abbreviations: SSTR2A, somatostatin receptor 2A; HIF, hypoxia-induced factor; NUC, nuclear; PPGL, pheochromocytoma and paraganglioma; Ki-67 LI, Ki-67 labeling index; MIBG, Metaiodobenzylguanidine; SDHB, succinate dehydrogenase subunit B; GAPP, Grading of Adrenal Pheochromocytoma and Paraganglioma.

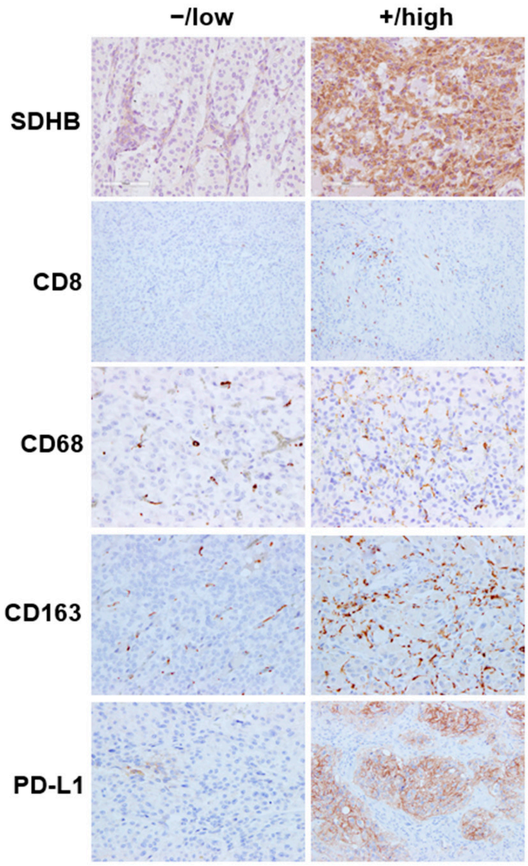

Figure S2. Representative immunohistochemistry staining images of SDHB, CD8, CD68, CD163, and PD-L1 in PPGLs (Magnification: ×400, except for CD8 at ×200). Abbreviations: SDHB, succinate dehydrogenase subunit B; PD-L1, programmed cell death ligand 1.

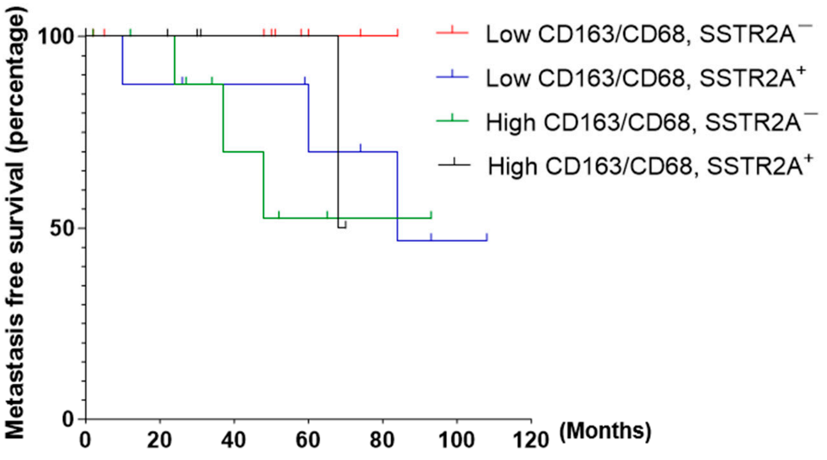

<u>Number at risk</u>

	0	20	40	60	80	100
Low CD163/CD68, SSTR2A⁻	11	7	7	3	1	0
Low CD163/CD68, SSTR2A⁺	8	7	6	5	3	1
High CD163/CD68, SSTR2A⁻	10	8	4	2	1	0
High CD163/CD68, SSTR2A⁺	5	6	2	2	0	0

Figure S3. The metastatic free survival analyses according to CD163/CD68 ratio and SSTR2A expression in patients with PPGLs. Patients with distant metastases at the time of primary tumor diagnosis were excluded from the analysis. Abbreviations: SSTR, somatostatin receptor; PPGL, pheochromocytoma, and paraganglioma.

Table S4. Characteristics of PD-L1 expression and CD163/CD68 ratio in PPGLs.

	PD-L1			CD163/CD68 ratio		
	Positive (n = 17)	Negative (n = 28)	*P*	high (n=23)	low (n=22)	*P*
**Age at Initial Diagnosis (median, range)**	41 (28-80)	52 (17-78)	0.314	46 (17-78)	43 (27-80)	0.928
**Sex**						
Female	10	18	0.759	14	14	1
Male	7	10		9	8	
**Primary Tumor Location**						
Adrenal	6	12	0.733	10	8	0.948
Extra-adrenal	11	16		13	14	
Abdominal	9	11		10	10	
Head and neck	1	1		1	1	
Bladder	1	4		2	3	
**Tumor Site**						
Adrenal	6	11	0.872	9	8	1
Abdominal	8	11		9	10	
Liver	1	0		1	0	
Head and neck	1	2		2	1	
Bladder	1	4		2	3	
**Functional Status**						
Adrenergic	6	7	0.173	7	6	0.918
Noradrenergic	2	10		7	5	
Silent or Dopaminergic	6	10		7	9	
Not available	3	1		2	2	
**Metastatic PPGL**						
Yes	6	12	0.757	12	6	0.13

No	11	16		11	16	
**Ki-67 LI**						
≥ 3%	9	14	1	13	10	0.556
< 3%	8	14		10	12	
**GAPP Score**						
≥ 7	3	9	0.563	8	4	0.296
3-7	11	16		11	16	
< 3	3	3		4	2	
**123I-MIBG Uptake**						
Positive	12	21	1	18	15	0.689
Negative	3	4		3	4	
Not available	2	3		2	3	
**SDHB Staining**						
Positive	11	21	0.511	14	18	0.189
Negative	6	7		9	4	

Abbreviations: PPGL, pheochromocytoma and paraganglioma; PD-L1, programmed cell death ligand 1; Ki-67 LI, Ki-67 labeling index; MIBG, Metaiodobenzylguanidine; SDHB, succinate dehydrogenase subunit B; GAPP, Grading of Adrenal Pheochromocytoma and Paraganglioma.

Table S5. Characteristics and group comparisons of CD163/CD68 ratio and SSTR2A expression in PPGLs.

	SSTR2A Negative, Low CD163/CD68 (n = 11)	SSTR2A Negative, High CD163/CD68 (n = 13)	SSTR2A Positive, Low CD163/CD68 (n=11)	SSTR2A Positive, High CD163/CD68 (n=10)	*P*
**Age at Initial Diagnosis (median, range)**	62 (39-80)	50 (18-78)	36 (27-70)	45.5 (17-59)	0.016
**Sex**					
Female	7	6	7	8	0.302
Male	4	7	4	2	
**Primary Tumor Location**					
Adrenal	5	6	3	4	0.949
Extra-adrenal	6	7	8	6	
Abdominal	4	6	6	4	
Head and neck	0	0	1	1	
Bladder	2	1	1	1	
**Tumor Site**					
Adrenal	5	5	3	4	0.357
Abdominal	4	5	6	4	
**Liver**	0	1	0	0	
Head and neck	0	1	1	1	
Bladder	2	1	1	1	
**Functional Status**					
Adrenergic	3	4	3	3	0.357
Noradrenergic	2	3	3	4	
Silent or Dopaminergic	5	4	4	3	
Not available	1	2	1	0	
**Metastatic PPGL**					
Yes	0	6	6	6	0.017
No	11	7	5	4	
**Ki-67 LI**					
≥ 3%	3	5	7	8	0.061

< 3%	8	8	4	2	
**GAPP Score**					
≥ 7	0	5	4	3	0.197
3-7	9	5	7	6	
< 3	2	3	0	1	
**123I-MIBG Uptake**					
Positive	9	10	6	8	0.005
Negative	1	1	3	2	
Not available	1	2	2	0	
**SDHB Staining**					
Positive	11	9	7	5	0.073
Negative	0	4	4	5	

Abbreviations: SSTR2A, somatostatin receptor 2A; HIF, hypoxia-induced factor; NUC, nuclear; PPGL, pheochromocytoma and paraganglioma; Ki-67 LI, Ki-67 labeling index; MIBG, Metaiodobenzylguanidine; SDHB, succinate dehydrogenase subunit B; GAPP, Grading of Adrenal Pheochromocytoma and Paraganglioma.
